# Efficacy and safety of adjunctive corticosteroids therapy for patients with severe community-acquired pneumonia: A systematic review and meta-analysis

**DOI:** 10.1097/MD.0000000000014636

**Published:** 2019-03-15

**Authors:** Jing Huang, Jiquan Guo, Hongtao Li, Weibin Huang, Tiantuo Zhang

**Affiliations:** a Department of Respiratory Medicine, The Third Affiliated Hospital of Sun Yat-sen University; b Institute of Respiratory Diseases, Sun Yat-sen University; c Department of Respiratory Medicine, Guangdong General Hospital, Guangzhou, China.

**Keywords:** corticosteroid, meta-analysis, severe community-acquired pneumonia

## Abstract

**Background:**

The systemic use of corticosteroids for patients in severe community-acquired pneumonia (CAP) remains disputed in clinical practice. We undertook a systematic review and meta-analysis to assess the efficacy and safety of corticosteroids in patients with severe CAP.

**Methods:**

We searched MEDLINE (1946 to June 2018), EMBASE (1966 to June 2018), and the Cochrane Library database for randomized controlled trials (RCTs) conducted for severe CAP. The endpoints of the study included total mortality, length of intensive care unit (ICU) stay and mechanical ventilation.

**Results:**

Nine trials which contained 914 patients were included for final meta-analysis. Of the 488 patients in the corticosteroid group, there were 37 deaths (7.58%) and 56 deaths occurred in 426 patients in the control group (13.1%). Corticosteroid therapy was associated with a lower rate of all-cause mortality compared to control (odd ratio [OR] 0.63, 95% confidence interval [CI] 0.42–0.95, *P* = .03). Subgroup analysis was conducted to show that the drug type modified the effect of steroids for mortality rate: prednisolone or methylprednisolone therapy (OR 0.37, 95% CI 0.19–0.72) reduced total mortality, whereas hydrocortisone use did not (OR 0.90, 95% CI 0.54–1.49). We found the length of ICU stay was significantly shorter in the steroid group compared to control (MD −2.52 days, 95% CI −4.88 to −0.15; *P* = .04). And there was a reduction trend in the need for mechanical ventilation in corticosteroid group (OR 0.53, 95% CI 0.28–1.02; *P* = .06). There was no trend towards more adverse events in the corticosteroid arm compared to control (OR 0.92, 95% CI 0.58–1.47; *P* = .74).

**Conclusion:**

Overall, adjunctive systemic corticosteroids therapy was effective and safe for patients with severe CAP. In addition, the effects of mortality may differ according to the type of corticosteroids.

## 1 Introduction

Community-acquired pneumonia (CAP) is a leading cause of morbidity and mortality worldwide.^[1–3]^ The mortality of severe CAP is reported to rise to 20%∼50%.^[2,4]^ Despite the developments in antimicrobial therapy and life-support measures, no substantial progress has been made in the last decades. Adjunct therapeutic interventions along with antibiotics may improve outcome of patients with severe CAP.

Corticosteroids, which attenuate the systemic inflammatory process in the disease process, are generally used as adjunctive agents.^[5,6]^ However, use of corticosteroids for these patients with severe pneumonia remains disputed in clinical practice. British guidelines state that “... steroids are not recommended in the routine treatment of high severity CAP”.^[7]^ While South African guidelines recommend “use of systemic corticosteroids should be considered in patients with severe CAP requiring intensive care unit (ICU) admission”.^[8]^ Several systematic reviews have explored the efficacy of corticosteroid in treatment of patients with severe CAP; however, these overviews were tested a few years ago or lacked appropriate subgroup analysis.^[9–11]^ There are still unanswered questions regarding which patients with severe CAP are most likely to benefit, which type to use, at what dose and for how long.^[12]^

The aim of this systematic review and meta-analysis of published randomized controlled clinical trials was to evaluate the efficacy and safety of systemic corticosteroid therapy in patients with severe CAP.

## 2 Methods

### 2.1 Date sources and search strategy

Relevant studies were identified by searching the following data sources: MEDLINE by OVID (from 1950 to June 2018), Embase (from 1970 to June 2018) and the Cochrane Library database (Cochrane Central Register of Active controlled Trials; no date restriction). Databases were searched using the following keywords

“severe community-acquired pneumonia”, “corticosteroid”, “steroid”, “prednisone”, “survival”, “adverse effects”, and “randomized controlled trials”. Trials were considered without language restrictions. Severe pneumonia was defined by the current clinical guidelines from British Thoracic Society (BTS), American Thoracic Society (ATS) or Pneumonia Severity Index (PSI) ≥4. As the data included in our study were extracted from published literature, no ethical approval and patient consent were required.

### 2.2 Data extraction and quality assessment

Relevant information was extracted into a spreadsheet. The extracted data included study characteristics (study name, country, and recruitment period), follow-up time, sample size, corticosteroids type, corticosteroids dose, and corticosteroids duration. The endpoints of the study included: total mortality, length of ICU stay, mechanical ventilation. Adverse events included hyperglycemia, gastrointestinal bleeding, and adverse cardiac events. And quality assessment of included randomized controlled trials (RCTs) was judged according to the Cochrane Collaboration tool for assessing the risk of bias.^[13]^ A literature review was done by 2 independent authors. When disagreement occurred, a third investigator was involved until consensus was reached.

### 2.3 Statistical analysis

We calculated odd ratio (OR) and 95% confidence interval (CI) for categorical variable by the random-effects model. For continuous outcomes, the difference in means and their 95% CI were calculated for each study, and the weighted mean difference was used as a summary estimate. I^2^ statistic was used to describe the percentage of variability that was due to heterogeneity beyond chance. And we analyzed publication bias using Begg and Egger tests. A 2-sided *P* <.05 was considered statistically significant, and all statistical analyses were performed using STATA, version 12.0 and Review Manager 5.0.

## 3 Results

### 3.1 Trial flow and study characteristics

**Figure 1:**
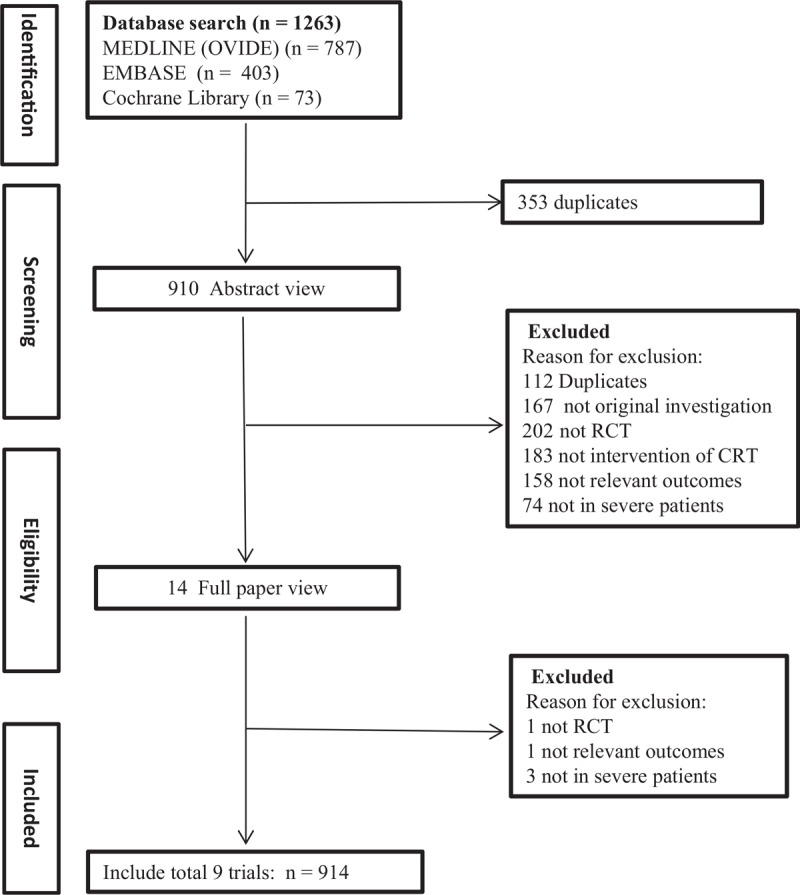
Process for identifying studies eligible for the meta-analysis.

The literature search yielded 1263 results for relevant articles, of which 14 were reviewed in full text. After a thorough and careful review, 9 trials which contained 914 patients were included for final meta-analysis (Fig. 1).^[14–22]^ The number of patients included ranged from 45 to 386. Among included RCTs, 4 studies included severe CAP patients which were defined by PSI of IV or V,^[16–18,22]^ 4 studies included patients defined according to guidelines from ATS,^[15,19–21]^ and 1 diagnosed according to BTS guidelines.^[14]^ The intervention included hydrocortisone in 5 studies,^[14,15,20–22]^ prednisone in 2 studies,^[16,18]^ and methylprednisolone in 2 studies.^[3,17]^ The duration of corticosteroid treatment was 10 days in 1 trial,^[17]^ 7 days in 6 trials,^[15,16,18,20–22]^ 5 days in 1 trial,^[19]^ and 1 day in 1 trial.^[14]^ Most trials used hydrocortisone dosage of 200 mg per day. Detailed extractive information from included studies was presented in Table 1.

**Table 1:**
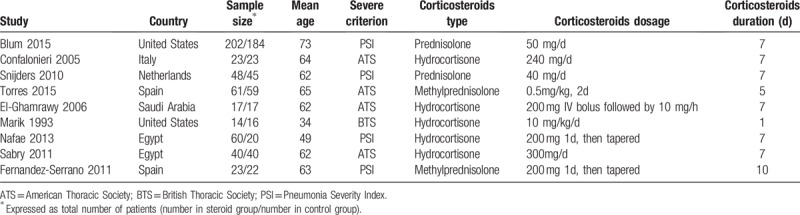
Characteristics of patients with baseline of included studies.

### 3.2 Quality assessment

**Figure 2:**
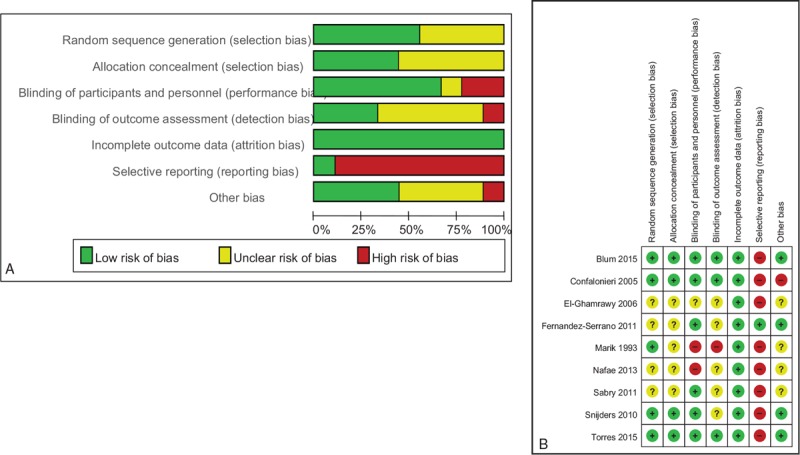
Risk of bias graph (A) and risk of bias summary (B).

We evaluated the quality of each study by sequence generation, allocation concealment, performance bias, detection bias, incomplete outcome data, selective reporting, and other possible sources of bias. The summary of the risk of bias is presented in Figure 2.

### 3.3 Total mortality

**Figure 3:**
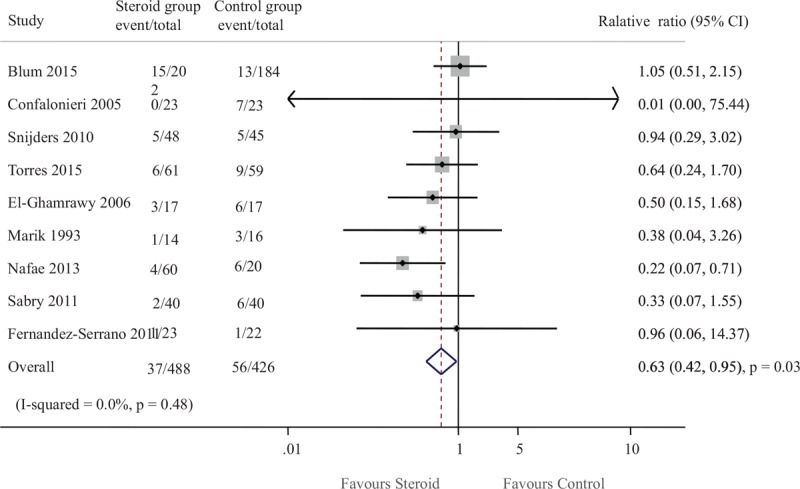
Effect of steroids on mortality in patients with severe CAP. CAP = community-acquired pneumonia.

Total mortality was reported in nine studies. Of the 488 patients in the corticosteroid group there were 37 deaths (7.58%) and 56 deaths occurred in 426 patients in the control group (13.1%). Corticosteroid therapy was associated with a lower rate of all-cause mortality compared to control (OR 0.63, 95% CI 0.42–0.95, *P* = .03) with no evidence of heterogeneity (I^2^ = 0.0%, *P* = .58) (Fig. 3).

**Figure 4:**
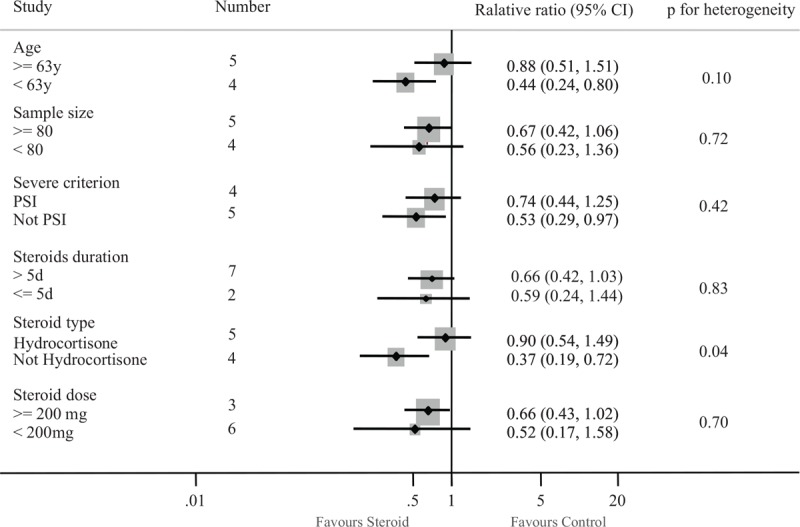
Subgroup analysis for the effect of steroids on mortality.

Furthermore, we explored the questions which patients with severe CAP are most likely to benefit, which steroid type to use, at what dose and for how long. Subgroup analyses were performed for total mortality (Fig. 4). We noted a different magnitude of effect according to the steroid type used in trials; the OR was 0.90 (95% CI 0.54–1.49) for a hydrocortisone regimen compared with 0.37 (95% CI 0.19–0.72) for prednisolone or methylprednisolone therapy (*P* for heterogeneity = .04). No clear evidence of heterogeneity was found in comparisons of summary results obtained from subsets of studies grouped by age, sample size, severe criterion, steroids duration, and steroid dose (Fig. 4).

### 3.4 Length of ICU stay

**Figure 5:**
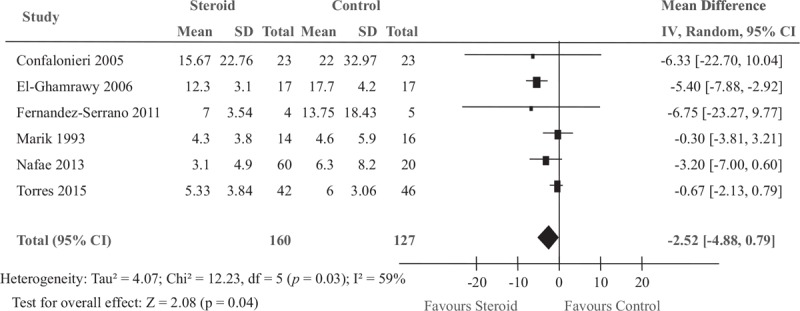
Effect of steroids on the length of ICU stay in patients with severe CAP. CAP = community-acquired pneumonia, ICU = intensive care unit.

Six studies included 287 patients who were admitted to the ICU. We found that length of ICU stay was significantly shorter in the steroid group compared to control (MD −2.52 days, 95% CI −4.88 to −0.15; *P* = .04) (Fig. 5).

### 3.5 Mechanical ventilation

**Figure 6:**
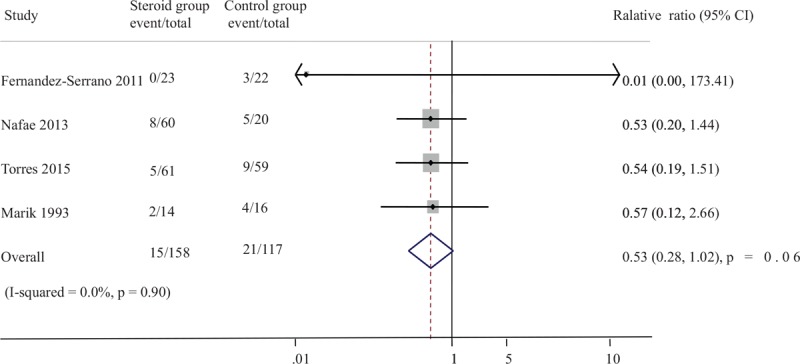
Effect of steroids on need for mechanical ventilation in patients with severe CAP. CAP = community-acquired pneumonia.

Four studies including 275 participants provided data regarding mechanical ventilation. There was a trend towards a reduction in the need for mechanical ventilation in the corticosteroid arm compared to control, but with borderline statistical significance (OR 0.53, 95% CI 0.28–1.02; *P* = .06) (Fig. 6).

### 3.6 Adverse events

Data on adverse events potentially associated with corticosteroid were collected from 6 trials (405 participants). Adverse events such as hyperglycemia, gastrointestinal bleeding and adverse cardiac events reported in the included studies were provided in Table 2. There was no trend towards more adverse events in the corticosteroid arm compared to control (OR 0.92, 95% CI 0.58–1.47; *P* = .74). The results showed that steroid therapy didn’t increase the rates of hyperglycemia (3 trials; OR 1.00, 95% CI 0.60–1.67, *P* = .99), gastrointestinal hemorrhage (6 trials; OR 1.00, 95% CI 0.38–2.60, *P* = .99), and adverse cardiac events (3 trials; OR 0.59, 95% CI 0.18–1.90, *P* = .38) (Table 2).

**Table 2:**
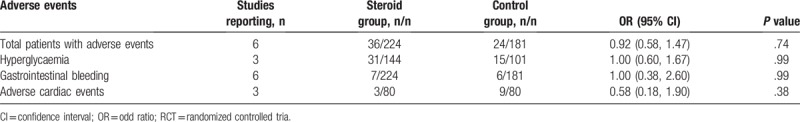
Adverse events reported in the included RCTs.

### 3.7 Sensitivity analysis

**Figure 7:**
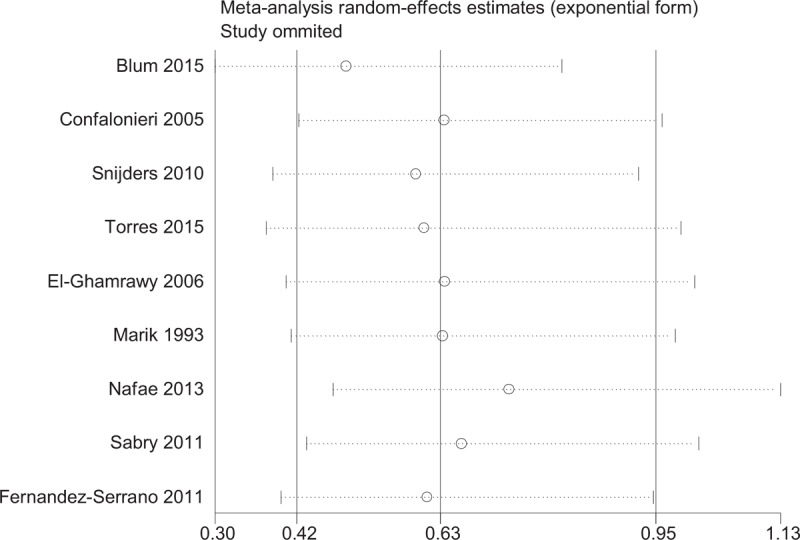
Sensitivity analyses of steroids and mortality.

The sensitivity analysis was performed by the sequential deletion of any individual article to measure the effects of each individual study. The results showed that the overall HRs were not significantly influenced by individual study (Fig. 7).

### 3.8 Publication bias

**Figure 8:**
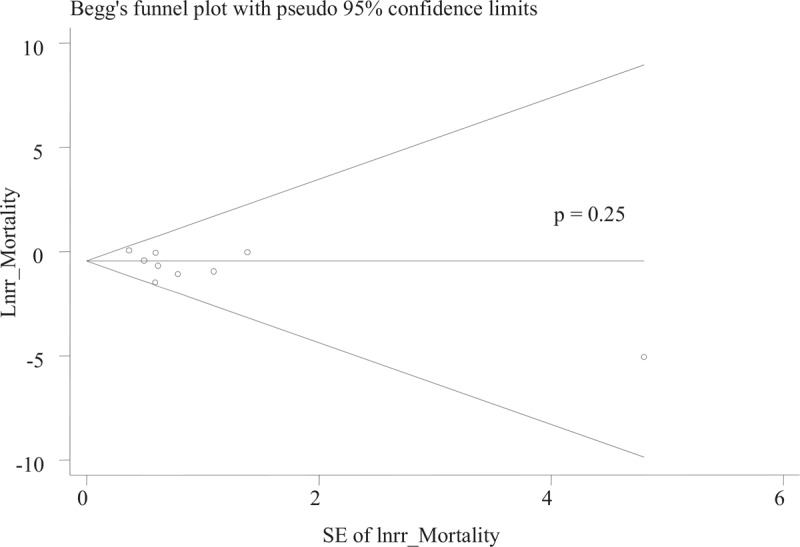
Forest plot for evaluation of publication bias for mortality.

Funnel plot of Egger test was used to show evidence of the publication bias and showed no significant publication bias regarding total mortality rate among studies (Egger test, *P* = .25, Fig. 8).

## 4 Discussion

The efficacy and safety for steroids therapy in patients with severe CAP remains controversial. This large quantitative review, comprising 9 trials and 914 individuals, has clearly demonstrated that steroid therapy reduced mortality rate in patients with severe CAP. Additionally, our analysis demonstrated that short-term application of steroid therapy didn’t increase the risk of adverse events.

Given the threat posed by severe CAP are linked to inflammation-mediated tissue damage and organ dysfunction, the use of steroid to reduce mortality is well founded.^[4,23]^ Several studies have provided high-quality evidence that steroid therapy could reduce total mortality in patients with severe CAP.^[15,24]^ Some studies did not show these beneficial effects of the addition of steroid therapy.^[14,17,18]^ The specific efficacy of corticosteroids therapy for patients with severe CAP has been evaluated by many other review groups. The conclusions of this study consistently support a trend towards favorable mortality impact on adults with severe CAP treated with steroids, which reduced the rate of mortality outcomes by 37% in compared with control group. There is potential reason for the survival advantage. The pathogenesis of the majority of severe CAP is usually closely related to the excessive inflammatory response. It is believed that corticosteroids, which play an important role in switching on genes that encode anti-inflammatory cytokines and switching off genes that encode pro-inflammatory cytokines, have anti-inflammatory effects.^[11,12]^ The question remains as to whom this benefit applies: who are the patients that will gain from steroid therapy? The reviews published in recent years do not answer these questions. Results of subgroup analysis showed that drug type modified the effect of steroids for mortality rate: prednisolone or methylprednisolone therapy reduced total mortality, whereas hydrocortisone use did not. Marik et al demonstrated that low-dose hydrocortisone does not affect serum TNF-a levels in patients with severe pneumonia.^[14]^ Another reason lies in steroids may work through different mechanisms. The association between steroid and risk of mortality was not modified by steroid dose or duration. Steroid therapy in severe CAP also reduced the length of ICU stay and the need for mechanical ventilation. Therefore, studies with large samples are strongly recommended to confirm the effect of prednisolone or methylprednisolone therapy on mortality.

Safety is an important concern with the use of steroid therapy in patients with severe CAP. Several studies have demonstrated there is an increased risk of pneumonia from taking regular inhaled steroids in patients with COPD.^[25]^ However, treatment of systemic steroids in severe CAP was not associated with an increased risk of adverse events in this study. Perhaps different administration routes may not have the same risk-benefit ratio in these patients. We noted that the incidences of hyperglycemia, gastrointestinal hemorrhage, and adverse cardiac events were not increased in the steroid group. Because the therapy is inexpensive and appears to be mostly well tolerated, it offers great promise as a method of substantially reducing the mortality rate and the length of ICU stay in patients with severe pneumonia. However, few trials reported the incidence of infection even though this was the most disconcerting side effect. Corticosteroids treatment can make it possible for patients to catch influenza, which is a cause of severe CAP. Indeed some experts do recommend excluding influenza infection before beginning corticosteroids therapy in severe CAP. Therefore, it must be used cautiously for these effective drugs.

Strengths of this meta-analysis were the rigorous methodology used. However, our study also has the following limitations. First, the corticosteroids regimens for severe CAP were not fully clarified; there was a little difference of the prescription of corticosteroids in these studies. Second, the definition of severe CAP was not consistent with these studies, which would affect the severity of disease and complications each study subsequently reported. Therefore, diagnosis of severe CAP in clinical practices should be comprehensive.

## 5 Conclusion

Overall, adjunctive corticosteroids therapy was effective and safe for patients with severe CAP. In addition, the effects of mortality may differ according to the type of corticosteroids. The clinical significance of the results requires confirmation with further high-quality RCTs.

## Acknowledgments

We thank all authors whose publications could be included in our meta-analysis.

## Author contributions

Jing Huang designed the study. Hongtao Li, Weibin Huang, and Tiantuo Zhang collected the data. Jiquan Guo analyzed the results, Jing Huang and Jiquan Guo drafted the manuscript.

**Conceptualization:** Jing Huang.

**Data curation:** HongTao Li.

**Formal analysis:** TianTuo Zhang.

**Methodology:** WeiBin Huang.

**Writing – original draft:** Jing Huang, Jiquan Guo.

**Writing – review & editing:** Jing Huang.

## References

[1] Woodhead M Welch CA Harrison DA Community-acquired pneumonia on the intensive care unit: secondary analysis of 17,869 cases in the ICNARC Case Mix Programme Database. Crit Care 2006;10 suppl 2:S1. 10.1186/cc4927 PMC3226135 16934135

[2] Ewig S Birkner N Strauss R New perspectives on community-acquired pneumonia in 388 406 patients. Results from a nationwide mandatory performance measurement programme in healthcare quality. Thorax 2009;64:1062–9. 19454409 10.1136/thx.2008.109785 PMC2782114

[3] Thomas CP Ryan M Chapman JD Incidence and cost of pneumonia in medicare beneficiaries. Chest 2012;142:973–81. 22406959 10.1378/chest.11-1160

[4] Laterre PF Severe community acquired pneumonia update: mortality, mechanisms and medical intervention. Crit Care 2008;12 Suppl 6:S1. 10.1186/cc7024 PMC2607111 19105794

[5] Rhen T Cidlowski JA Antiinflammatory action of glucocorticoids--new mechanisms for old drugs. New Engl J Med 2005;353:1711–23. 16236742 10.1056/NEJMra050541

[6] Endeman H Meijvis SC Rijkers GT Systemic cytokine response in patients with community-acquired pneumonia. Eur Respir J 2011;37:1431–8. 20884746 10.1183/09031936.00074410

[7] Lim WS Baudouin SV George RC BTS guidelines for the management of community acquired pneumonia in adults: update 2009. Thorax 2009;64 suppl 3:iii1–55. 19783532 10.1136/thx.2009.121434

[8] Boyles TH Brink A Calligaro GL South African guideline for the management of community-acquired pneumonia in adults. J Thorac Dis 2017;9:1469–502. 28740661 10.21037/jtd.2017.05.31 PMC5506119

[9] Stern A Skalsky K Avni T Corticosteroids for pneumonia. Cochrane Database Syst Rev 2017;12:CD007720. 29236286 10.1002/14651858.CD007720.pub3 PMC6486210

[10] Wu WF Fang Q He GJ Efficacy of corticosteroid treatment for severe community-acquired pneumonia: a meta-analysis. Am J Emerg Med 2018;36:179–84. 28756034 10.1016/j.ajem.2017.07.050

[11] Bi J Yang J Wang Y Efficacy and safety of adjunctive corticosteroids therapy for severe community-acquired pneumonia in adults: an updated systematic review and meta-analysis. PLoS One 2016;11:e0165942. 27846240 10.1371/journal.pone.0165942 PMC5113003

[12] Feldman C Anderson R Corticosteroids in the adjunctive therapy of community-acquired pneumonia: an appraisal of recent meta-analyses of clinical trials. J Thorac Dis 2016;8:E162–71. 27076965 10.21037/jtd.2016.02.43 PMC4805802

[13] Higgins JP Altman DG Gotzsche PC The Cochrane Collaboration's tool for assessing risk of bias in randomised trials. BMJ 2011;343:d5928. 22008217 10.1136/bmj.d5928 PMC3196245

[14] Marik P Kraus P Sribante J Hydrocortisone and tumor necrosis factor in severe community-acquired pneumonia. A randomized controlled study. Chest 1993;104:389–92. 8339624 10.1378/chest.104.2.389

[15] Confalonieri M Urbino R Potena A Hydrocortisone infusion for severe community-acquired pneumonia: a preliminary randomized study. Am J Respir Crit Care Med 2005;171:242–8. 15557131 10.1164/rccm.200406-808OC

[16] Snijders D Daniels JM de Graaff CS Efficacy of corticosteroids in community-acquired pneumonia: a randomized double-blinded clinical trial. Am J Respir Crit Care Med 2010;181:975–82. 20133929 10.1164/rccm.200905-0808OC

[17] Fernandez-Serrano S Dorca J Garcia-Vidal C Effect of corticosteroids on the clinical course of community-acquired pneumonia: a randomized controlled trial. Crit Care 2011;15:R96. 21406101 10.1186/cc10103 PMC3219361

[18] Blum CA Nigro N Briel M Adjunct prednisone therapy for patients with community-acquired pneumonia: a multicentre, double-blind, randomised, placebo-controlled trial. Lancet 2015;385:1511–8. 25608756 10.1016/S0140-6736(14)62447-8

[19] Torres A Sibila O Ferrer M Effect of corticosteroids on treatment failure among hospitalized patients with severe community-acquired pneumonia and high inflammatory response: a randomized clinical trial. JAMA 2015;313:677–86. 25688779 10.1001/jama.2015.88

[20] El-Ghamrawy AH SM Esmat AA Effects of low-dose hydrocortisone in ICU patients with severe community-acquired pneumonia. Egyptian J Chest 2006;55:91–9.

[21] Sabry NAOE Corticosteroids and ICU course of community acquired pneumonia in Egyptian settings. Pharmacol Pharm 2011;2:73–81.

[22] Nafae RM RM Amany FM Rashed SB Adjuvant role of corticosteroids in the treatment of community-acquired pneumonia. Egyptian J Chest Dis Tuberculosis 2013;62:439.

[23] Steinberg KP Hudson LD Goodman RB Efficacy and safety of corticosteroids for persistent acute respiratory distress syndrome. New Engl J Med 2006;354:1671–84. 16625008 10.1056/NEJMoa051693

[24] Stern A Leibovici L Paul M Corticosteroids reduce mortality in patients with severe community acquired pneumonia (CAP). Clin Infect Dis Off Publ Infect Dis Soc Am 2018. 10.1093/cid/ciy336 29897416

[25] Crim C Dransfield MT Bourbeau J Pneumonia risk with inhaled fluticasone furoate and vilanterol compared with vilanterol alone in patients with COPD. Ann Am Thorac Soc 2015;12:27–34. 25490706 10.1513/AnnalsATS.201409-413OC

